# Prospective cross-sectional study on faecal immunochemical tests: sex specific cut-off values to obtain equal sensitivity for colorectal cancer?

**DOI:** 10.1186/s12876-014-0217-7

**Published:** 2014-12-21

**Authors:** Sietze T van Turenhout, Frank A Oort, René WM van der Hulst, Arjen P Visscher, Jochim S Terhaar sive Droste, Pieter Scholten, Anneke A Bouman, Gerrit A Meijer, Chris JJ Mulder, Leo GM van Rossum, Veerle MH Coupé

**Affiliations:** Department of Gastroenterology and Hepatology, VU University Medical Centre, Amsterdam, the Netherlands; Gastroenterology and Hepatology, Kennemer Gasthuis, Haarlem, the Netherlands; Gastroenterology and Hepatology, Sint Lucas Andreas Hospital, Amsterdam, the Netherlands; Clinical Chemistry, VU University Medical Centre, Amsterdam, the Netherlands; Pathology, VU University Medical Centre, Amsterdam, the Netherlands; Department for health evidence, Radboud University Medical Centre, Nijmegen, the Netherlands; Epidemiology and Biostatistics, VU University Medical Centre, Amsterdam, the Netherlands

**Keywords:** Colorectal cancer screening, Advanced adenoma, Fecal immunochemical test, Sex

## Abstract

**Background:**

Faecal immunochemical tests (FITs) are commonly used in colorectal cancer (CRC) screening. Diagnostic accuracy of FIT differs between males and females. This so far unexplained difference could result in a dissimilarity in screening outcome between both sexes. The aim of this study is to compare sensitivity and specificity of a FIT between males and females, and study potential explanatory variables.

**Methods:**

In this cross-sectional study, data were prospectively collected. 3,022 subjects performed a FIT prior to complete colonoscopy. Sensitivity, specificity, and ROC curves were compared for both sexes. Potential explanatory variables of the relation between sensitivity and sex were explored.

**Results:**

At all cut-off values, FIT sensitivity for CRC was higher (range 13-23%) and specificity was lower (range 2-4%) in males compared to females. At 75 ng/ml, sensitivity for CRC was 93% in males compared to 71% in females (p = 0.03), and specificity was 90% in males compared to 93% in females (p = <0.05). For advanced adenomas, males had a slightly higher sensitivity and lower specificity (not significant). At 75 ng/ml, sensitivity for advanced adenomas was 33% in males compared to 29% in females (p = 0.46), and specificity was 93% in males compared to 95% in females (p = 0.22). ROC curves were similar for both sexes, and equal combinations of sensitivity and specificity could be achieved by adjusting the cut-off values. For CRC, the difference in sensitivity could not be explained by age or location of the tumour.

**Conclusions:**

FIT has a higher sensitivity and a lower specificity for CRC in males than in females. Equal test characteristics can be achieved by allowing separate cut-off values for both sexes. Location and age do not explain the observed differences in sensitivity.

**Electronic supplementary material:**

The online version of this article (doi:10.1186/s12876-014-0217-7) contains supplementary material, which is available to authorized users.

## Background

Colorectal cancer (CRC) is a leading cause of cancer related death worldwide [1]. Early detection by population screening is the most realistic approach to reduce CRC-related death. Faecal immunochemical tests (FITs) are increasingly used as the primary screening test for CRC [2].

Recently, it was found that FITs have a higher sensitivity and lower specificity for advanced colorectal neoplasia in males compared to females [3]. Although diagnostic accuracy of a screening test is just one of several factors determining the efficiency of a screening programme, a difference in FIT characteristics between males and females could implicate disparities in the expected benefit from CRC screening. Such a difference may require tailored screening. Whether this difference reflects, for example, a dissimilarity in advanced neoplasia distribution or a difference in the age of onset of CRC, remains to be resolved.

The aim of the present study is to investigate whether the sensitivity and specificity of a frequently used FIT for the detection of CRC and advanced adenomas differs between males and females, and whether this difference can be explained by age, location, number and/or size of neoplastic lesions.

## Methods

### Study population

For the current analysis, data were used from an ongoing study programme on FIT performance. This programme aims to answer several research questions and has been previously described extensively [4–6]. In short, individuals scheduled for elective colonoscopy in 5 participating medical centres were invited to participate and perform a FIT prior to colonoscopy. In addition to the exclusion criteria of these previous studies, individuals with an indication of visible rectal bleeding or anaemia were excluded from the analysis to minimize potential work-up bias. Use of NSAIDs and/or aspirin was no exclusion criterium, and subjects were not advised on whether to continue this medication on the day of colonoscopy. The study was approved by the VU University Medical Centre Ethics Committee, Kennemergasthuis Ethics Committee, Sint Lucas Andreas Ethics Committee, Zaans Medical Centre Ethics Committee and Slotervaart Hospital Ethics Committee. All participants provided written informed consent.

### Study design

In this cross-sectional study, data were collected prospectively. The test used was an automated FIT with quantitative results: OC-sensor® (Eiken Chemical Co., Tokyo, Japan). One experienced technician performed the analyses, and was kept unaware of the clinical data. All tests were analysed by using the OC sensor MICRO desktop analyser (Eiken Chemical co, Tokyo, Japan) [7]. A cut-off of ≥75 ng/ml is advised by the manufacturer. Here, haemoglobin concentrations of ≥50, ≥75, ≥100, and ≥200 ng/ml of buffer solution were taken as cut-off values. These concentrations correspond to faecal haemoglobin concentrations of respectively ≥10, ≥15, ≥20 and ≥40 milligram of haemoglobin per gram of faeces.

### Colonoscopy and lesions

Experienced gastroenterologists performed or supervised all colonoscopies. The endoscopists were unaware of the FIT result, in order to prevent investigator bias. Conscious sedation by midazolam was offered to all patients.

A colonoscopy was considered complete when the caecum was intubated with identification of the appendicle orifice and valvula Bauhini, or when an obstructing neoplasm was found. Quality control measures included documentation of colonic landmarks. Individuals in whom the bowel cleansing was insufficient, and individuals in whom the colonoscopy remained incomplete were excluded from analysis. However, if a barium enema, virtual colonography or second colonoscopy was performed within six months, evaluation of the colon was considered complete and the subject was included in analysis. The right colon was defined as the proximal part of the colon including caecum, ascending colon, right (or hepatic) flexure and transverse colon. The left colon was defined as the distal part of the colon including left (or splenic) flexure, descending colon, sigmoid and rectum [8,9]. In case of multiple neoplasia detected on colonoscopy, patients were classified based on the most advanced lesion found.

Tissue samples obtained at colonoscopy were sent to the department of pathology and evaluated according to current standards. Adenomas ≥1.0 cm, adenomas with a villous component (i.e. tubulovillous or villous adenoma) or adenomas with severe/high-grade dysplasia were classified as advanced adenomas [10,11].

### Statistical analysis

Using colonoscopy as the reference test, sensitivities and specificities of FIT were calculated for two definitions of colonoscopy outcome: (i) the presence of CRC and (ii) the presence of advanced adenoma. Sensitivity was defined as the proportion of positive test results in patients with the colonoscopy outcome under consideration. Specificity was calculated as the proportion of negative test results in patients with an outcome less severe than the outcome under consideration.

Sensitivity and specificity of FIT at different cut-off values were compared between males and females using the Fisher Exact test. Analyses were repeated for the symptomatic and screening/surveillance indication groups separately. Receiver Operating Characteristic (ROC) curves were plotted for both sexes separately and the Areas Under the ROC Curves (AUC’s) were calculated, along with their 95% confidence intervals. The 95% confidence intervals were used to compare diagnostic capabilities.

To study whether sex dissimilarities are a reflection of differences in age, location and size or number of neoplastic lesions, a stratified analysis of 2×2 contingency tables was performed, with statistical testing using the Fishers Exact test. Age was dichotomized into subjects <65 and 65 or older. Location was divided in left and right sided lesions. The number of advanced adenomas was grouped as 1 or >1, and size was grouped <10 mm or ≥10 mm.

In addition, the effect of these covariates on the relation between test sensitivity (for CRC and advanced adenoma) and sex was studied in a multivariate logistic regression analysis. In this analysis, we aimed to estimate the independent effect of each factor after adjusting for the contributions of other variables in the model. Full multivariate models (i.e., forced entry with no variable selection) were used, because it was of interest to study the extent to which the coefficient of sex would be influenced when including other covariates in the model. The Wald test was used to detect significant factors in multivariate logistic regression analysis models. Age was used as continuous variable. To avoid dilution of the potential independent effect of location on FIT results, subjects with CRC on both sides of the colon, and subjects with advanced adenomas on both sides of the colon were excluded from the multivariate analysis. Stratified analyses and the multivariate analysis were performed for a FIT cut-off value of 50, 75 and 100 ng/ml. The manuscript contains the results at the cut-off value of 75 ng/ml. In the supplementary data, the results of multivariate logistic regression analysis for CRC and advanced adenomas are shown for the cut-off values of 50 and 100 ng/ml. The variables studied for CRC and advanced adenomas were sex, age, and location of the lesion. Additionally, T-stage and presence of advanced adenomas were used as variables for predicting sensitivity for CRC. For advanced adenomas, the size and number of advanced adenomas were added to the analysis.

There was no correction of p-values to recognize that several tests of statistical significance were performed on data arising from individual patients, because the purpose of the research was to highlight any potential differences.

All analyses were performed with SPSS for Windows Version 20 (SPSS Inc., Chicago, USA).

## Results

### Participants

**Figure 1:**
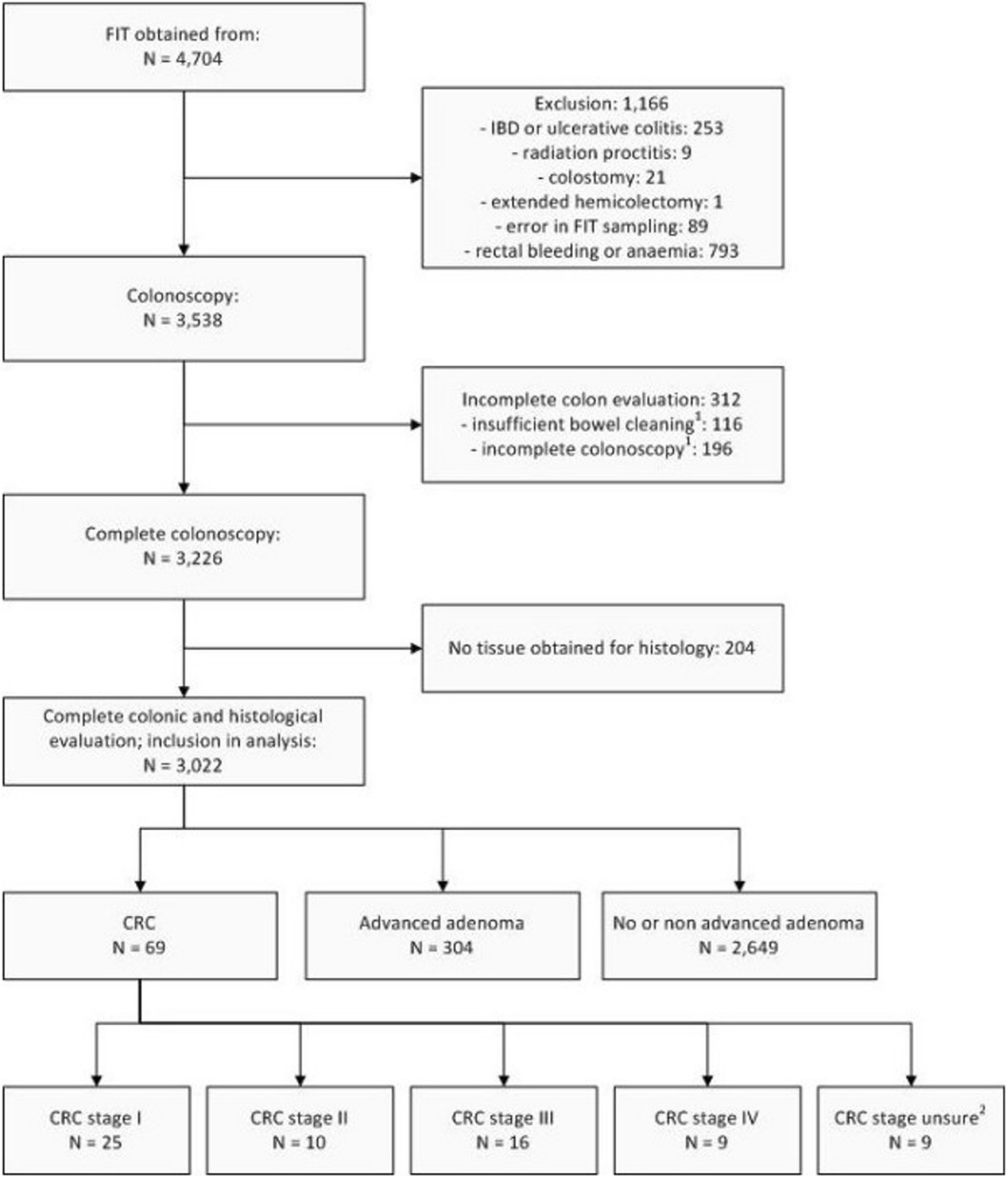
**Study flow diagram.** ^1^Incomplete colon evaluation in spite of possible additional evaluation by repeated colonoscopy, barium enema or virtual colonography within 6 months. ^2^CRC stage remained unsure as these cases received pre-operative radiotherapy or due to disseminated disease exact classification remained unknown.

Between June 2006 and October 2010, 4,704 subjects returned a FIT and underwent colonoscopy. Of these, 1,682 were excluded for different reasons (see Figure 1), leaving 3,022 participants for analysis. As the first colonoscopy was incomplete in 107 participants, a second colonoscopy, barium enema or CT-colonography was needed to complete the colonoscopic evaluation. The mean age of the participants was 59.7 years (range 19–91 years, SD 12.6), and 45% was male. The indication for colonoscopy was evaluation of symptoms in 44% (1,331/3,022), screening or surveillance in 47% (1,412/3,022), and unspecified in 9% (279/3,022) (see Table 1). Characteristics of included males and females are shown in Table 2.

**Table 1:** **Primary indication for colonoscopy among 3,022 consecutive patients included for analysis of FIT characteristics according to sex**

**Indication group**	**Indication for colonoscopy**	**N**
**Symptomatic**	Weight loss	88
	Clinical suspicion of diverticulitis	26
	Clinical suspicion of IBD	40
	Abdominal pain	355
	Altered bowel habits	548
	Clinical or radiological suspicion of CRC*	54
	Diarrhoea	128
	Constipation	92
	**Total**	**1,331**
**Screening & surveillance**	Average risk	103
	Familial history of CRC	482
	Lynch syndrome	54
	Polyp surveillance	578
	Post CRC surveillance	195
	**Total**	**1,412**
**Other**	Not specified/others	**279**
**Grand total**		**3,022**

*This group includes individuals who were referred for colonoscopy as CRC was suspected on abdominal sonography, CT/MRI scan or physical examination.

FIT = faecal immunochemical test, IBD = inflammatory bowel disease, CRC = colorectal cancer.

**Table 2:** **Characteristics of males and females with CRC and advanced adenomas included in comparative analysis of a FIT test**

	**Males**	**Females**
**Total number of subjects**	**1,312**	**1,641**
**CRC**	**N = 45**	**N = 24**
Mean age	68.0 years	68.8 years
Positive tests*	93%	71%
Location (left sided/right sided/missing)	67%/31%/2%	71%/29%/0%
Symptomatic/screening & surveillance/unknown	64%/18%/18%	67%/25%/8%
**Advanced Adenoma**	**N = 164**	**N = 140**
Mean age	63.9 years	64.3 years
Positive tests*	33%	29%
Location (left sided/right sided/missing)	55%/36%/9%	64%/29%/7%
Number of advanced adenomas >1	21%	16%
Size of advanced adenoma >9 mm	70%	66%
Symptomatic/screening & surveillance/unknown	28%/54%/18%	38%/45%/17%

*Cut-off value 75 ng/ml. CRC = colorectal cancer; FIT = faecal immunochemical test.

### Colonoscopy

In 2.3% of the included subjects, CRC was found (69/3,022) and in another 10.1% one or more advanced adenomas were detected (304/3,022; see Figure 1). From all subjects with CRC, 11.6% (8/69) also had one or more advanced adenomas. Males were found to have a higher prevalence of CRC (45/1357 = 3.3%) than females (24/1665 = 1.4%; p = 0.001). The distribution of CRC stages according to the TNM classification, is demonstrated in Figure 1. The T-stage (Tumour-stage) distribution was 16% (11/69) T1, 25% (17/69) T2, 33% (23/69) T3 and 6% (4/69) T4. In 14 (20%) individuals with CRC, T-stage was unknown due to preoperative radiotherapy, or was not determined because of disseminated disease at time of diagnosis. The prevalence of T1/T2 and T3/T4 tumours was comparable in males (17/32 = 53%) and females (11/23 = 48%; p = 0.70).

### Positivity rates

For the total population, the FIT positivity rate was 12.3% and 12.7% at a cut-off level of 50 and 75 ng/ml, respectively. The positivity rate decreased with increasing cut-off values, to 6.8% at 200 ng/ml. Males were found to have a higher positivity rate (50 ng/ml; 15.3%, 75 ng/ml; 12.7%) compared to females (50 ng/ml; 9.8%, 75 ng/ml; 8.3%, p-values < 0.001).

### Sensitivity and specificity

In the total study population, the sensitivity for CRC at a cut-off value of 50 and 75 ng/ml was 88% and 86%, respectively. Specificity was 90% and 92% at 50 and 75 ng/ml. For advanced adenomas, sensitivity and specificity were 35% and 92% at 50 ng/ml and 31% and 94% at 75 ng/ml, respectively (see Table 3). At all cut-off values, males were found to have a higher sensitivity for CRC than females. The difference in sensitivity ranged from 13% to 23% and was largest and significant at the cut-offs 75 and 100 ng/ml. More specifically, at 75 ng/ml, sensitivity for CRC was 93% in males compared to 71% in females (p = 0.03). The specificity for CRC was significantly lower in males compared to females, but the difference was small (between 2.2 and 3.9%, see Table 3). For advanced adenomas, the differences in sensitivity and specificity between males and females showed the same pattern as for CRC. However, the higher sensitivity in males was small (not significant, see Table 3). Only specificity of FIT in males at a cut-off value of 50 ng/ml was significantly lower than in female participants (see Table 3).

**Table 3:** **Comparison of sensitivity and specificity of a FIT for detection of CRC and advanced adenomas in males and females at different cut-off values**

**CRC**						**Advanced adenoma**					
**Sensitivity**						**Sensitivity**					
**FIT cut-off**	Total N = 69*	Males N = 45*	Females N = 24*	**Difference**	**p-value**	**FIT cut-off**	**Total N = 304** ^**†**^	**Males N = 164** ^**†**^	**Females N = 140** ^**†**^	**Difference**	**p-value**
50 ng/ml (CI)	88.4% (78–95)	93.3% (82–99)	79.2% (58–93)	14.1%	0.12	50 ng/ml (CI)	34.9% (30–41)	36.6% (39–45)	32.9% (25–41)	3.7%	0.55
75 ng/ml (CI)	85.5% (75–93)	93.3% (82–99)	70.8% (49–87)	22.5%	**0.03**	75 ng/ml (CI)	30.9% (26–37)	32.9% (26–41)	28.6% (21–37)	4.3%	0.46
100 ng/ml (CI)	85.5% (75–93)	93.3% (82–99)	70.8% (49–87)	22.5%	**0.03**	100 ng/ml (CI)	28.6% (24–34)	31.7% (25–39)	25.0% (18–33)	6.7%	0.21
200 ng/ml (CI)	75.4% (64–85)	80.0% (65–90)	66.7% (45–84)	13.3%	0.25	200 ng/ml (CI)	21.1% (17–26)	24.4% (18–32)	17.1% (11–24)	7.3%	0.16
**Specificity**						**Specificity**					
**FIT cut-off**	**Total N = 2,953** ^**#**^	**Males N = 1,312** ^**#**^	**Females N = 1,641** ^**#**^	**Difference**	**p-value**	**FIT cut-off**	**Total N = 2,649** ^**‡**^	**Males N = 1,148** ^**‡**^	**Females N = 1,501** ^**‡**^	**Difference**	**p-value**
50 ng/ml (CI)	89.5%(88–91)	87.3% (85–89)	91.2% (90–93)	−3,9%	**<0,05**	50 ng/ml (CI)	92.3% (91–93)	90.8% (89–92)	93.4% (92–95)	−2.6%	**<0.05**
75 ng/ml (CI)	91.5% (90–95)	90.1% (88–92)	92.6% (91–94)	−2,5%	**<0,05**	75 ng/ml (CI)	94.0% (93–95)	93.4% (92–95)	94.5% (93–96)	−1.1%	0.22
100 ng/ml (CI)	92.6% (92–94)	91.2% (90–93)	93.7% (92–95)	−2,5%	**<0,05**	100 ng/ml (CI)	95.0% (94–96)	94.4% (93–96)	95.5% (94–97)	−1.1%	0.24
200 ng/ml (CI)	94.8% (94–96)	93.6% (21–95)	95.8% (95–97)	−2,2%	**<0,05**	200 ng/ml (CI)	96.6% (96–97)	96.2% (95–97)	97.0% (96–98)	−0.8%	0.28

*This concerns the total of subjects with CRC from which the sensitivity was calculated. ^#^This concerns the total of subjects without CRC from which the specificity was calculated. ^†^This concerns the total of subjects with advanced adenoma from which the sensitivity was calculated. ^‡^This concerns the total of subjects without CRC and without advanced adenomas from which the specificity was calculated. FIT = faecal immunochemical test; CRC = colorectal cancer; CI = confidence interval.

Results for the symptomatic and screening/surveillance indication groups separately are in line with results for the total population. In the group with symptomatic individuals, sensitivity for CRC at 75 ng/ml was 93% in males and 81% in females. In the screening/surveillance group these figures were 100% and 50% respectively. However, the screening/surveillance group consisted of only 14 carcinomas. The specificity for CRC at 75 ng/ml in the symptomatic group was in 92% males and 92% in females. In the screening/surveillance group these figures were 90% and 93% respectively. For advanced adenomas, sensitivity at 75 ng/ml in the symptomatic group was 26% in males and in 32% females. These figures were respectively 33% and 24% in the screening/surveillance group. In the symptomatic group, specificity for advanced adenomas at 75 ng/ml was 94% in males and also 94% in females. For the screening/surveillance group these figures were 93% and 95% respectively.

### Receiver operating characteristic curves

**Figure 2:**
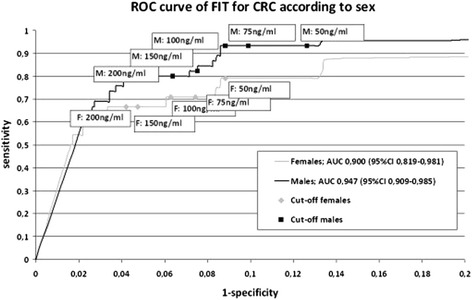
**Receiver operating characteristic curve of FIT for detection of CRC.** ROC = receiver operating characteristic; FIT = faecal immunochemical test; CRC = colorectal cancer; AUC = area under the curve*.*

**Figure 3:**
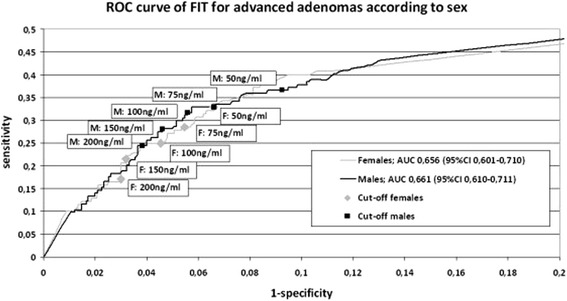
**Receiver operating characteristic curve of FIT for detection of advanced adenoma.** ROC = receiver operating characteristic FIT = faecal immunochemical test; AUC = area under the curve.

The test characteristics for males and females at each cut-off value are visualized in the ROC curves in Figures 2 and 3. The AUC’s for CRC for males and females were 0.95 (95% CI 0.909-0.985) and 0.90 (95% CI 0.819-0.981) respectively. The ROC curves and AUC’s for advanced adenoma were very similar between males and females (see Figure 3).

### The influence of potential explanatory variables

In patients with CRC, males were found to have a higher FIT positivity rate than females (50 ng/ml 93% vs. 79%, p = 0.08). This difference persisted when stratifying CRC by location (100% vs. 88% (left-sided) and 79% vs. 51% (right-sided)). Overall for males and females, left sided CRC was found to have a higher positivity rate (96%) than right sided CRC (71%, p < 0.05). However, within males and females with CRC, an equal proportion of cancers was left sided. In multivariate analysis, location and sex were found to be associated with FIT sensitivity for CRC (see Table 4 and Additional file 1: Tables S1 and S2 for cut-off values 50 and 100 ng/ml). The univariate odds ratio for sex (OR 5.8, 95% CI 1.3-25.0) was only mildly affected by the inclusion of location and age in the analysis (OR 6.2, 95% CI 1.2-32.9). As such, the relation between sex and FIT sensitivity is not a result of differences in age of the patients or location of the tumour between males and females.

**Table 4:** **Level of significance and odds ratio’s for different variables used in a multivariate logistic regression model predicting sensitivity of a FIT at cut-off of 75 ng/ml for detection of CRC and advanced adenomas**

	**CRC**					**Advanced adenoma**			
	**Univariate model**		**Multivariate model**			**Univariate model**		**Multivariate model**	
**Variable**	**OR (95% CI)**	**p-value**	**OR (95% CI)**	**p-value**	**Variable**	**OR (95% CI)**	**p-value**	**OR (95% CI)**	**p-value**
Sex (ref = female) (N = 69)	**5.77 (1.33-24.95)**	**0.02**	**6.16 (1.15-32.92)**	**0.03**	Sex (ref = female) (N = 304)	1.23 (0.75-2.00)	0.41	1.00 (0.57-1.75)	0.99
Age (continuous variable) (N = 69)	1.01 (0.94-1.08)	0.89	1.00 (0.92-1.08)	0.96	Age (continuous variable) (N = 304)	1.01 (0.98-1.03)	0.55	1.00 (0.98-1.03)	0.75
Location (ref = right-sided) (N = 68)	**4.30 (1.07-17.35)**	**0.04**	**4.83 (0.95-24.8)**	**0.05**	Location (ref = right-sdided (N = 303)	**1.94 (1.26-2.98)**	**0.002**	1.31 (0.79-2.20)	0.30
T-stage (ref = T1) (N = 55)	1.57 (0.39-6.32)	0.53	2.19 (0.44-10.80)	0.34	AA size (ref = <10 mm) (N = 285)	**5.58 (2.55-12.22)**	**<0.001**	**5.24 (2.32-11.83)**	**<0.001**
Presence of AA (ref = none) (N = 69)	0.83 (0.09-7.54)	0.87	1.38 (0.09-20.52)	0.81	Number of AA (ref = 1) (N = 300)	**4.82 (2.63-8.85)**	**<0.001**	**4.27 (2.10-8.70)**	**<0.001**

FIT = faecal immunochemical test; CRC = colorectal cancer; AA = advanced adenoma; OR = odds ratio; CI = confidence interval.

In individuals with advanced adenomas, males were found to have a slightly higher positivity rate than females (37% vs. 33%, n.s.). Left sided advanced adenomas were more frequently positive (38%) than right sided lesions (24%, p = 0.02). Also, subjects with more than one advanced adenoma were found to have a higher FIT positivity rate (63%) than subjects with one advanced adenoma (27%, p < 0.001). The same was found for subjects with an advanced adenoma >9 mm (45%) compared to advanced adenomas <10 mm (12%, p < 0.001).

In males, a slightly higher proportion of advanced adenomas was right-sided (40% in males versus 32% in females) and was large in size (76% in males versus 71% in females). In addition, in men multiple synchronous advanced adenomas were more common (21% in males versus 17% in females, not significant). The distribution of these explanatory variables caused a shift in the OR for sex from 1.2 in the univariate analysis to 1.0 in the multivariate analysis, indicating that the small difference in sensitivity between males and females can be explained by differences in the size and number of advanced adenomas (see Table 4 and Additional file 1: Tables S1 and S2). In conclusion, the size and the number of advanced adenomas were important predictors of FIT positivity in advanced adenomas, but sex was not.

## Discussion

In the present study, sensitivity and specificity of a frequently used FIT was assessed in males and females in a large cohort of subjects referred for colonoscopy. FIT was found to be more sensitive and less specific for CRC in men than in women. The areas under the ROC curves were similar for males and females. By using different cut-off values for both sexes, a similar sensitivity can be reached. The difference in sensitivity between the sexes could not be explained by age or location of the lesion. The sensitivity of FIT for advanced adenomas was slightly higher in males (not significant) and strongly related to size and the number of advanced adenomas.

In males, FIT was found to have 13-23% higher sensitivity for CRC than in females. Previous studies already found a difference in test performance for the detection of advanced neoplasia in males and females [3]. In the German study, positive and negative predictive values, as well as sensitivity and specificity were calculated. However, due to the inclusion of only 14 cases of CRC, test characteristics could not be calculated for CRC. The current study not only included a higher number of advanced adenomas, it also evaluated the relation between sex and FIT sensitivity for CRC, corrected for the location of colonic lesions and age of the participant. These variables were chosen because it is known that CRC develops earlier in lifetime in men then in women [12]. In addition, it is also known that females have a higher prevalence of right sided CRC [13]. Finally, left sided neoplasia have a higher likelihood to test positive on FIT [9,14]. A potential explanation for this phenomenon, could be a difference in degradation of haemoglobin between left and right sided lesions, or difference in shape and tendency to bleed between proximal and distal lesions [14].

In the multivariate analysis, location and sex were significantly associated with FIT sensitivity for CRC, but the univariate odds ratio for sex was not substantially affected by the inclusion of location and age in the analysis. By stratified analysis of 2×2 contingency tables, it was found that the observed relation between sex and FIT sensitivity for CRC could not be explained by either location or age. It can only be speculated on whether the sex difference is due to other confounders, such as tumour size, tumour biology (e.g. blood vessel density), or colon transit time, which is known to be longer in females [15]. Other authors hypothesized that the higher serum concentration of haemoglobin in male blood could cause higher FIT positivity when blood is lost in the colon [3]. This cannot be confirmed in the current study, as the serum haemoglobin level of participants was not determined. For advanced adenomas, no significant difference in sensitivity was found between the sexes, but males consistently had a higher sensitivity. In multivariate analysis and stratified 2×2 contingency tables, it was found that the number and size of lesions are predictive for test sensitivity. This last observation is in line with another study that showed that the number and size of polyps influence test accuracy for detection of advanced adenomas [16].

Currently, CRC screening with preselection by means of FIT sampling is a one size fits all approach. Sex specific screening guidelines could be considered, in order to optimize the effectiveness of a screening programme in both males and females [3,17]. Based on the current results, the lower sensitivity of FIT in women could lead to a smaller benefit from screening for females. However, the lower sensitivity of FIT in females may be counterbalanced by a higher participation rate, as was found in the English and Scottish screening programmes [18,19]. Therefore, multiple factors need to be taken into account determine the efficiency of a CRC screening programme. When preferred, the same sensitivity for CRC could be easily reached by lowering the cut-off value in females. In the absence of a sex difference in the sensitivity for advanced adenomas, sex specific cut-off values in screening seem unnecessary, as prevalent cancers will presumably be detected in the first screening rounds. In the next screening rounds the focus of screening may shift to detection of advanced adenomas. In addition, individualising screening guidelines adds to the complexity of a screening programme and should only be adopted if the expected benefits are substantial. Individualized screening guidelines may confuse providers and consumers to the point of decreasing adherence [20].

The current study provides insight into the relation between sex and the test characteristics of FIT for detection of CRC and advanced adenomas. To the best of our knowledge, this is the first study to observe that sex specific differences in FIT sensitivity are mainly present in CRC and only small in advanced adenomas. Each participant in the study underwent complete colonoscopy regardless of FIT outcome. This enabled direct calculation of not only sensitivity but specificity as well. The high number of advanced colonic neoplasia in the referral population that was used, enabled us to stratify for CRC, which was not possible before [3].

For proper interpretation of the results, some limitations need to be discussed. The main limitation is that a heterogeneous population was studied. That is, both symptomatic subjects and subjects with higher than average risk for CRC (e.g. derived from adenoma and CRC surveillance programmes) were included. Consequently, it cannot be ruled out that, even after exclusion of subjects with anaemia and haematochezia, sensitivity may be overestimated and specificity underestimated due to work-up bias [21]. This may occur as symptomatic participants have an increased likelihood for having both a positive FIT and an advanced colorectal neoplasm. This is why the current study for instance did not aim to provide an optimal cut-off value for each sex in the screening setting. On the other hand, using non-screening study populations to investigate test characteristics in subgroups that are usually underrepresented in screening studies seems justified. This is supported by a previous study that showed that CRC cases from a non screening setting had similar results as CRC cases from a screening study [22]. Another limitation it that location of lesions was assessed by the endoscopists by recognition of colonic landmarks. Although this is the most commonly used method for assessment of the location, the accuracy with which the location of the neoplastic lesions can be determined may not be optimal. In addition, as size of CRC was not prospectively scored, it was not possible to use this variable in the logistic regression analysis. Finally, there may be other explanatory variables for FIT performance that were not included, like for instance the use of NSAIDs [9].

## Conclusions

In conclusion, males were found to have a higher FIT sensitivity and a lower specificity for CRC compared to females. However, as the shapes of and the areas under the ROC curves are similar, equal characteristics can be achieved by allowing different cut-off values for both sexes. Location of CRC and age of the individuals are not responsible for the observed differences in sensitivity. Whether the difference is relevant in screening remains questionable. No significant difference in test characteristics for advanced adenomas was found between the sexes.

## Additional file

Additional file 1:
**Supplementary data.**

## Abbreviations

AUC: Area under the curve
CI: Confidence interval
CRC: Colorectal cancer
FIT: Faecal immunochemical test
IBD: Inflammatory bowel disease
NSAIDs: Non Steroidal Anti Inflammatory Drugs
OR: Odds ratio
ROC: Receiver operating characteristic
T-stage: Tumor-stage
