# Supplementary material for: Prospective cross-sectional study on faecal immunochemical tests: sex specific cut-off values to obtain equal sensitivity for colorectal cancer?
Source: BMC Gastroenterol. 2014 Dec 21;14:217. doi: 10.1186/s12876-014-0217-7 (PMC4302436; doi:10.1186/s12876-014-0217-7)
Supplement: Additional file 1: — Supplementary data. [file 12876_2014_217_MOESM1_ESM.doc]

**Supplementary data**

The supplementary data contain two tables that show the results of multivariate logistic regression analysis on the effect of different variables in predicting sensitivity of a FIT for detection of colorectal cancer (CRC) and advanced adenomas. The multivariate analyses were performed for a FIT cut-off value of 50 and 100 ng/ml. The independent effect of each factor was investigated after adjusting for the contributions of other variables in the model. The variables studied for CRC and advanced adenomas were gender, age, and location of the lesion. Additionally, T-stage and presence of advanced adenomas were used as variables for predicting sensitivity for CRC. For advanced adenomas, the size and number of advanced adenomas were added to the analysis.

**Supplementary Table 1. Level of significance and odds ratio’s for different variables used in a multivariate logistic regression model predicting sensitivity of a FIT at cut-off of 50 ng/ml for detection of CRC and advanced adenomas.**

|  | **CRC** | | | |  | **Advanced adenoma** | | | |
| --- | --- | --- | --- | --- | --- | --- | --- | --- | --- |
|  | **Univariate model** | | **Multivariate model** | |  | **Univariate model** | | **Multivariate model** | |
| **Variable** | **OR**  **(95%CI)** | **p-value** | **OR**  **(95%CI)** | **p-value** | **Variable** | **OR (95%CI)** | **p-value** | **OR (95%CI)** | **p-value** |
| Sex  (ref = female)  (N = 69) | 3.7  (0.80-17.02) | 0.10 | 4.23  (0.75-24.65) | 0.10 | Sex  (ref = female)  (N = 304) | 1.2  (0.73-1.90) | 0.50 | 0.9  (0.54-1.62) | 0.82 |
| Age  (continuous variable)  (N = 69) | 1.02  (0.94-1.10) | 0,70 | 1.01  (0.91-1.12) | 0.88 | Age  (continuous variable)  (N = 304) | 1.0  (0.99-1.04) | 0.27 | 1.0  (0.98-1.04) | 0.49 |
| Location (ref = right-sided)  (N = 68) | **9.0**  **(1.64-49.45)** | **0.01** | **8.74**  **(1.35-56.64)** | **0.02** | Location (ref = right-sided)  (N = 303) | **2.0**  **(1.34-3.11)** | **<0.05** | 1.4  (0.84-2.33) | 0.19 |
| T-stage  (ref = T1)  (N = 55) | 0.96  (0.21-4.29) | 0.95 | 1.21  (0.22-6.79) | 0.83 | AA size  (ref = <10mm)  (N = 285) | **6.1**  **(2.87-12.79)** | **<0.05** | **5.7**  **(2.60-12.30)** | **<0.05** |
| Presence of AA  (ref = none)  (N = 69) | 1.10 | 0.93 | 1.00  (0.07-14.38) | 1.00 | Number of AA  (ref = 1)  (N = 300) | **4.6**  **(2.50-8.44)** | **<0.05** | **4.0**  **(1.95-8.16)** | **<0.05** |

*FIT = faecal immunochemical test; CRC = colorectal cancer; AA = advanced adenoma; OR = odds ratio; CI = confidence interval.*

**Supplementary Table 2. Level of significance and odds ratio’s for different variables used in a multivariate logistic regression model predicting sensitivity of a FIT at cut-off of 100 ng/ml for detection of CRC and advanced adenomas.**

|  | **CRC** | | | |  | **Advanced adenoma** | | | |
| --- | --- | --- | --- | --- | --- | --- | --- | --- | --- |
|  | **Univariate model** | | **Multivariate model** | |  | **Univariate model** | | **Multivariate model** | |
| **Variable** | **OR**  **(95%CI)** | **p-value** | **OR**  **(95%CI)** | **p-value** | **Variable** | **OR (95%CI)** | **p-value** | **OR (95%CI)** | **p-value** |
| Sex  (ref = female)  (N = 69) | **5.77**  **(1.33-24.95)** | **0.02** | **6.16**  **(1.15-32.92)** | **0.03** | Sex  (ref = female)  (N = 304) | 1.39  (0.84-2.31) | 0.20 | 1.18  (0.66-2.08) | 0.58 |
| Age  (continuous variable)  (N = 69) | 1.01  (0.94-1.08) | 0.89 | 1.00  (0.92-1.09) | 0.96 | Age  (continuous variable)  (N = 304) | 1.01  (0.99-1.03) | 0.45 | 1.01  (0.98-1.04) | 0.57 |
| Location (ref = right-sided)  (N = 68) | **4.30**  **(1.07-17.35)** | **0.04** | 4.83  (0.94-24.81) | 0.06 | Location (ref = right-sided)  (N = 303) | **1.93**  **(1.24-2.99)** | **<0.01** | 1.25  (0.74-2.10) | 0.40 |
| T-stage  (ref = T1)  (N = 55) | 1.57  (0.39-6.32) | 0.53 | 2.19  (0.44-10.80) | 0.34 | AA size  (ref = <10mm)  (N = 285) | **4.85**  **(2.21-10.63)** | **<0.01** | **4.38**  **(1.94-9.92)** | **<0.01** |
| Presence of AA  (ref = none)  (N = 69) | 0.83  (0.09-7.54) | 0.87 | 1.38  (0.09-20.52) | 0.81 | Number of AA  (ref = 1)  (N = 300) | **5.18**  **(2.81-9.53)** | **<0.01** | **4.61**  **(2.27-9.37)** | **<0.01** |

*FIT = faecal immunochemical test; CRC = colorectal cancer; AA = advanced adenoma; OR = odds ratio; CI = confidence interval.*
